# Radiculopathy due to cement extravasation after sacroplasty: A case report

**DOI:** 10.1016/j.inpm.2026.100808

**Published:** 2026-08-06

**Authors:** Daniel Wang, Ali Mostoufi

**Affiliations:** Department of Physical Medicine and Rehabilitation, Lynn Rehabilitation Hospital, University of Miami Miller School of Medicine, Miami, FL, USA

**Keywords:** Sacroplasty, Sacral insufficiency fracture, Osteoporosis, Cement extravasation, Radiculopathy, Vertebroplasty, Kyphoplasty, Transforaminal epidural steroid injection

## Abstract

Sacral insufficiency fractures are an underrecognized source of atraumatic lumbopelvic pain in elderly osteoporotic patients and may evade diagnosis when initial radiographs are inconclusive. We describe an elderly osteoporotic woman who presented to an outpatient spine clinic with atraumatic low back and hip girdle/groin pain, without radicular symptoms or neurologic deficit. After failure of four weeks of conservative treatment, MRI confirmed bilateral sacral insufficiency fractures, and CT-guided bilateral sacroplasty was performed for persistent axial pain. The procedure was complicated by immediate severe right L5–S1 radicular pain, with MRI and CT demonstrating cement extravasation into the right S1 neural foramen, posterior epidural space, cephalad to the L5 vertebral body, and caudally within the sacral canal. Conservative management with pharmacologic therapy, rehabilitation, and a single right L5 and S1 transforaminal epidural steroid injection resulted in gradual symptom resolution. Spine surgery was consulted, but operative intervention was deferred because symptoms improved and no neurologic deficit developed. This case highlights both the diagnostic complexity of sacral insufficiency fractures and the importance of prompt recognition of cement migration after sacroplasty, while supporting nonoperative management in selected patients without neurologic deficit.

## 1 Introduction

Sacral insufficiency fractures are an increasingly recognized cause of axial lumbosacral, pelvic, Sacral insufficiency fractures are an increasingly recognized cause of axial lumbosacral, pelvic, hip girdle, and groin pain in elderly and osteoporotic patients [[1], [2], [3]]. Despite growing awareness, these fractures remain underdiagnosed because of their nonspecific clinical presentation and the poor sensitivity of plain radiographs, particularly in the early stages [[2], [3], [4]]. Magnetic resonance imaging (MRI) is highly sensitive for identifying sacral insufficiency fractures and associated bone marrow edema when initial radiographs are nondiagnostic [[2], [3], [4]]

When conservative measures such as activity modification, physical therapy, and pharmacologic pain management fail to provide adequate symptom relief, image guided sacroplasty has emerged as a minimally invasive treatment option associated with rapid pain reduction and functional improvement [[5], [6], [7]]. Although sacroplasty is generally considered safe, cement extravasation remains the most commonly reported procedural complication [[6], [7], [8], [9]]. While most cases are detected radiographically and remain asymptomatic, extravasation into the neural foramina or epidural space can result in clinically significant radiculopathy, with pain, functional impairment, and neurologic symptoms [[8], [9], [10], [11]]

Management of symptomatic cement extravasation after sacroplasty remains incompletely defined [[6], [7], [8]].˒ [10] Surgical decompression may be warranted in the presence of progressive neurologic deficit or bowel or bladder dysfunction; however, reports of successful nonsurgical management in carefully selected patients are limited [[8], [9], [10], [11]]. We describe a patient who developed radiculopathy secondary to cement extravasation after sacroplasty and was successfully treated with conservative measures, including a targeted epidural steroid injection, without the need for surgery.

## 2 Case presentation

An elderly osteoporotic woman initially presented for consultation with atraumatic low back and hip girdle/groin pain rated 6/10, without associated leg pain, motor deficits, or bowel or bladder dysfunction. Symptoms persisted despite four weeks of conservative treatment, including physical therapy and pharmacologic management. Lumbar radiographs were inconclusive, showing only age-appropriate degenerative changes consistent with spondylosis. Subsequent MRI confirmed bilateral sacral insufficiency fractures corresponding to the patient's lumbosacral pain.

**Image 1:**
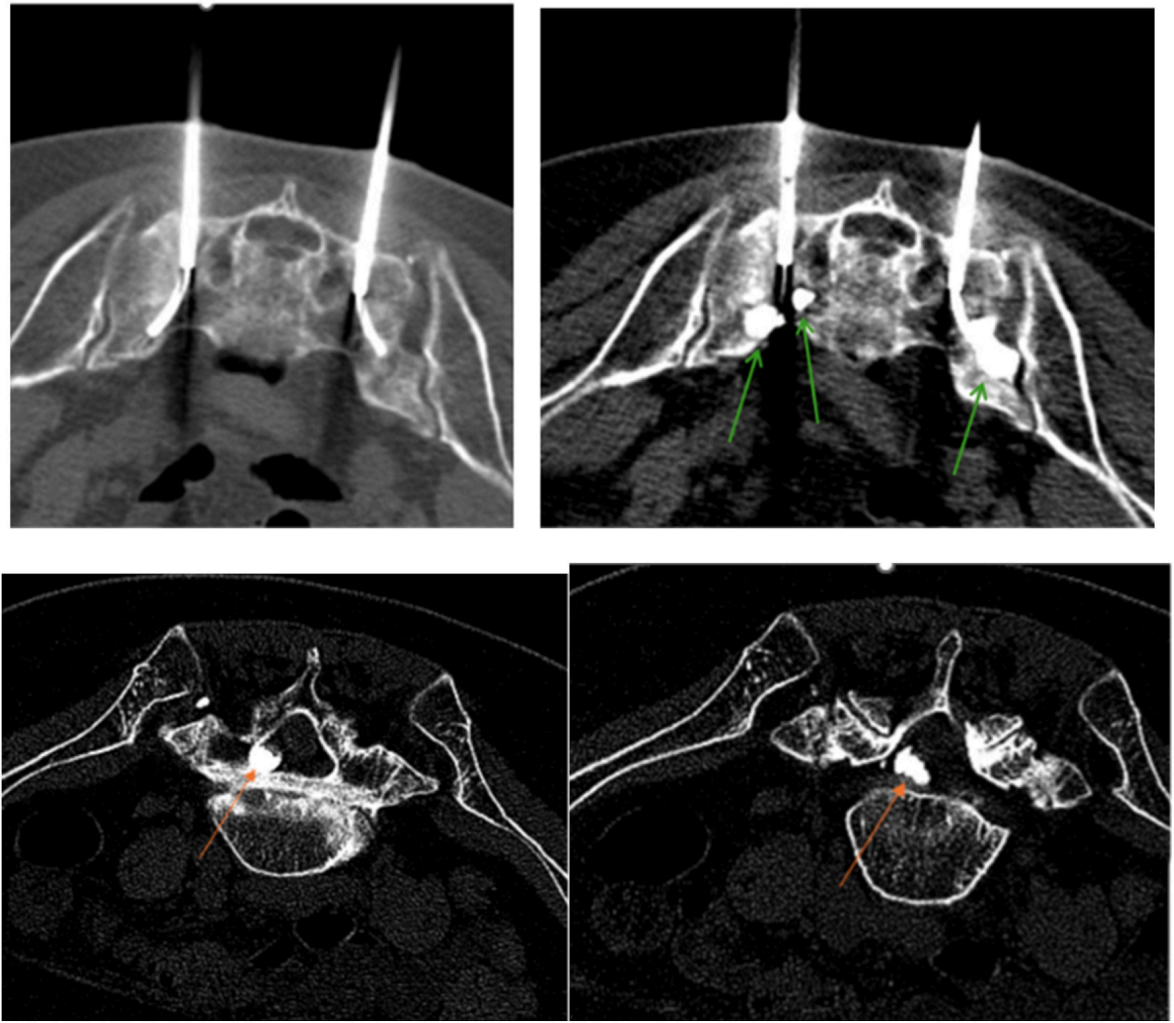
*Image 1. CT-guided sacroplasty of the sacrum. The image on the top row shows CT-guided left- and right-sided needle (cannula) placement for sacroplasty prior to cement injection. The image on the right shows cement injection during sacroplasty, with the left-sided needle showing lateral flow of polymethylmethacrylate within the sacrum. Cement delivered through the right-sided needle shows both medial and lateral dispersion, with medial cement extravasation extending toward adjacent neural structures.* The second-row images are post sacroplasty CT showing cement extravasation (arrow)posterior to L5 and S1 vertebrae within the spinal canal.

**Image 2:**
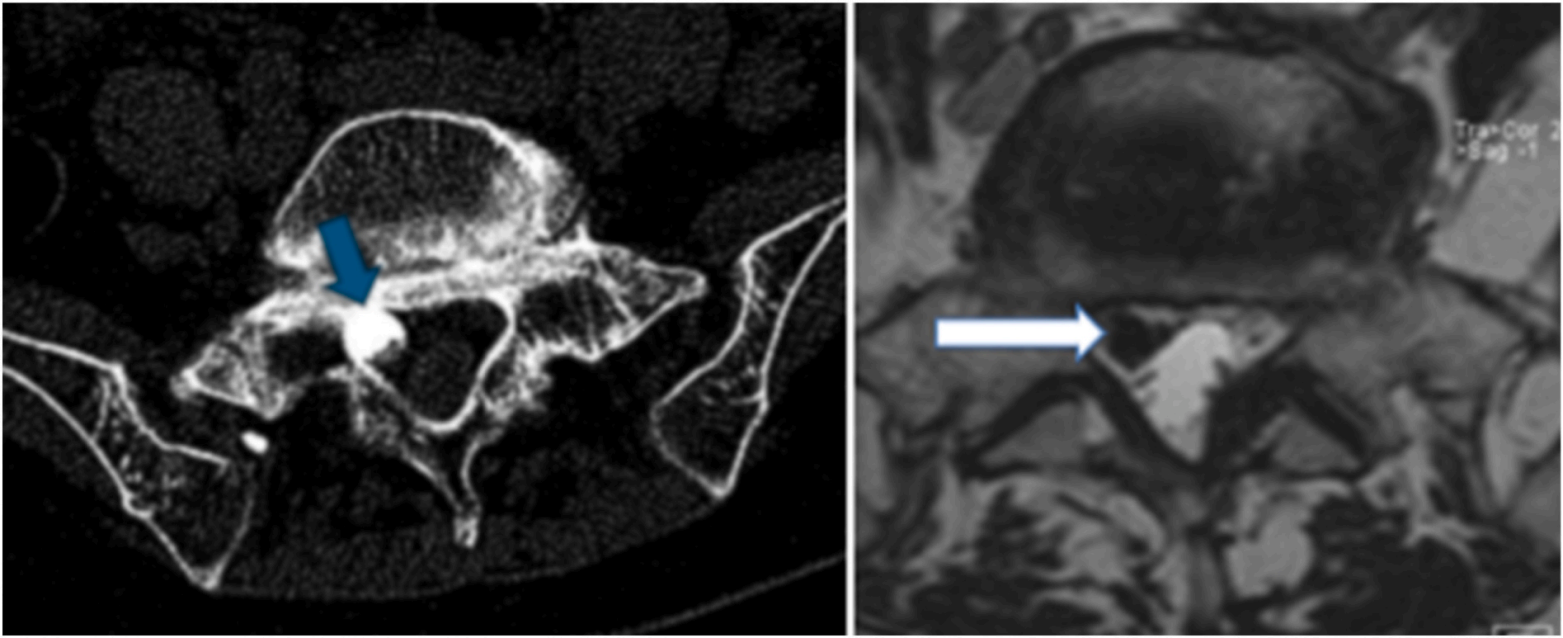
*Image 2. Correlation of cement extravasation and neural compression following sacroplasty.* The left image (axial CT, bone window) demonstrates polymethylmethacrylate cement extending beyond the intended sacral body into the right S1 neural foramen and adjacent epidural space (arrow). The right image (axial MRI) shows corresponding mass effect on the right-sided neural elements at the lumbosacral junction (arrow), providing radiographic correlation with the patient's acute post-procedural right L5–S1 radicular symptoms.

**Image 3:**
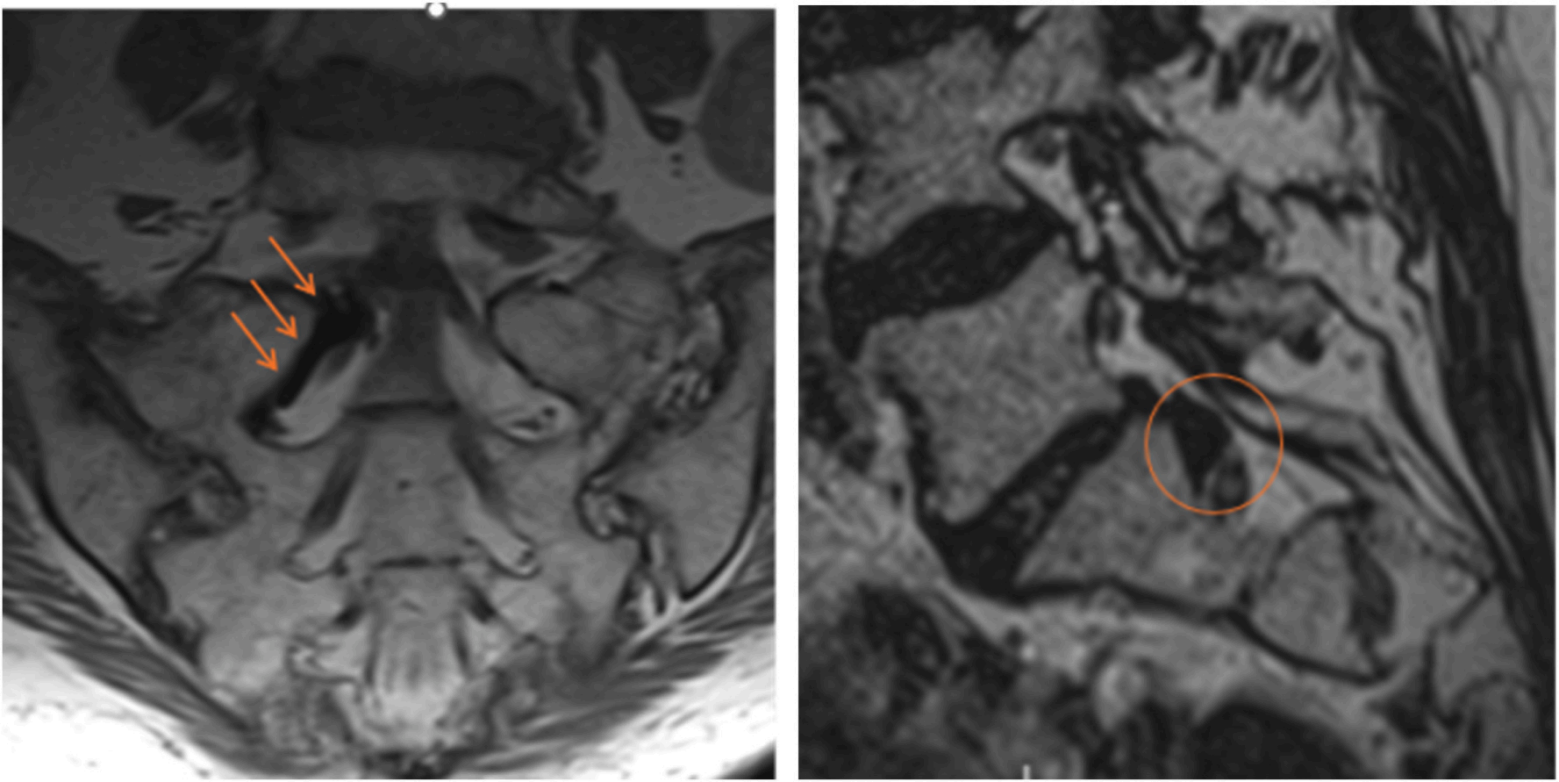
Image 3. Image on left shows *Coronal MRI view demonstrates polymethylmethacrylate cement extravasation with rostral extension toward the L5 level and intracanal extravasation along the expected course of the right S1 nerve root, correlating with the patient's post-procedural radicular symptoms. On the sagittal view, the cement is noted posterior to S1 vertebral body (image on right)*.

Given persistent pain and functional limitation despite conservative therapy, the patient underwent CT guided bilateral sacroplasty. Immediately after the procedure, she developed new onset severe right side radicular pain in the L5 and S1 distribution, with a reported VAS score of 8/10. Walking and standing tolerance became significantly limited because of the radicular pain, resulting in substantial functional impairment. Follow up MRI and CT showed cement extravasation into the right S1 neural foramen and posterior epidural space, extending cephalad to the L5 vertebral body and caudally within the sacral canal along the S1 nerve root (Image 1, Image 2, Image 3).

**Image 4:**
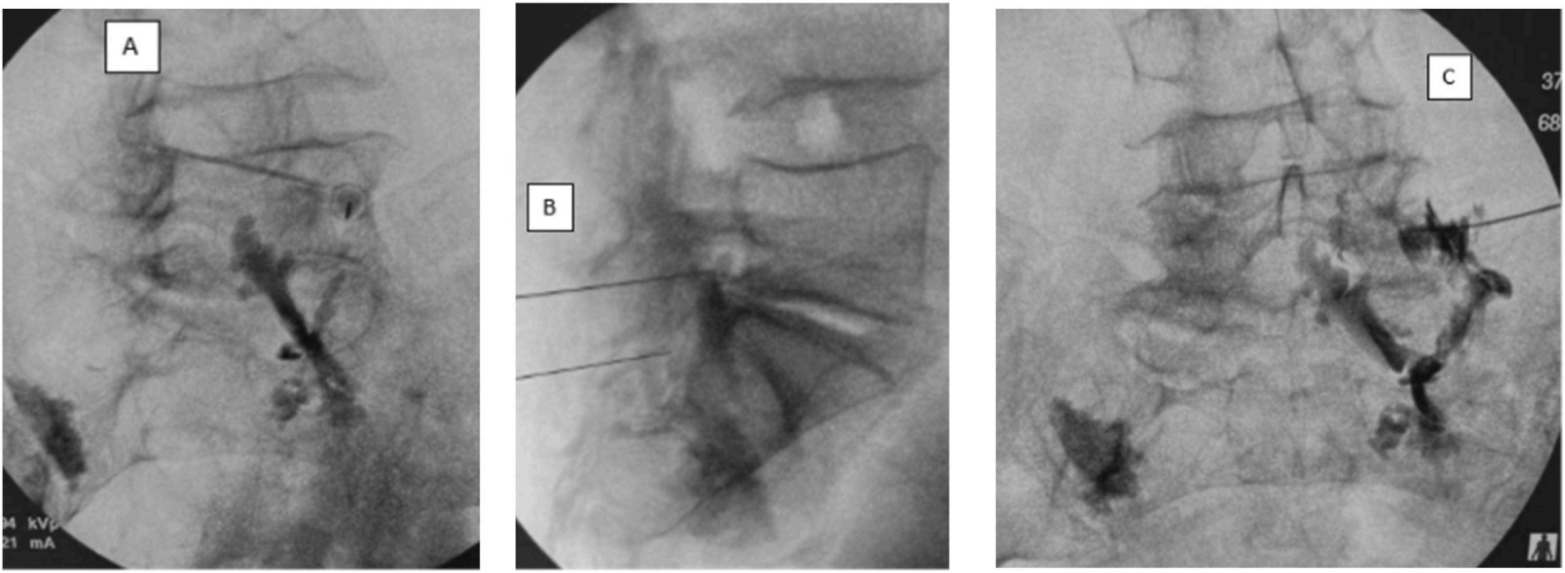
Images A and B show fluoroscopic needle guidance for Right L5 and S1 Transforaminal epidural followed by contrast injection (C). Prior to contrast injection (A and B), the dark material within right and left sacrum represents the cement from previous sacroplasty.

In the absence of progressive neurologic deficit or bowel or bladder dysfunction, the patient was managed conservatively with pharmacologic therapy, physical rehabilitation, and a single right L5 and S1 transforaminal epidural steroid injection (Image 4). This resulted in gradual improvement and near complete resolution of radicular symptoms. After the epidural injection, the patient reported a VAS score of 0 to 1/10, with walking and standing function returning to near baseline. The spine surgery team was consulted; however, surgical intervention was not recommended given the patient's clinical improvement and the absence of neurologic impairment.

This case highlights the importance of early recognition of cement migration after sacroplasty and supports conservative management in carefully selected patients without progressive neurologic deficit.

## 3 Discussion

Sacroplasty is a well-established minimally invasive treatment for sacral insufficiency fractures and has been associated with substantial pain relief and functional improvement in appropriately selected patients [[5], [6], [7]]. Cement extravasation remains the most frequently reported procedural complication, although it is typically asymptomatic and identified incidentally on postprocedural imaging [[6], [7], [8], [9]]. Recent sacroplasty specific imaging studies suggest that foraminal and epidural cement leakage may be associated with radicular symptoms, paralleling observations reported in the vertebroplasty literature [6,9,10].

Computed tomography (CT) guidance provides superior anatomic visualization and may facilitate earlier recognition of cement migration in high risk regions such as the neural foramina and sacral canal [[12], [13], [14]]. By contrast, fluoroscopy, although widely available and capable of real time imaging, may be limited in its ability to visualize posterior sacral anatomy in certain projections [11,13]. Accordingly, the choice of imaging modality should be individualized on the basis of patient anatomy, fracture morphology, and operator experience.

Prior case reports of symptomatic radiculopathy after sacroplasty have described variable management strategies depending on neurologic severity and the extent of neural compression on imaging. For example, Barber et al. reported a case of S1 radiculopathy caused by cement leakage completely encasing the nerve root on CT, which required surgical decompression and cement removal to achieve pain relief and functional recovery [15,16]

New dermatomal pain after sacroplasty should be considered pathologic until proven otherwise and warrants prompt cross-sectional imaging. Management of cement related radiculopathy should be guided by neurologic status, symptom severity, and imaging findings. Although conservative management may be effective in selected patients, surgical decompression is indicated in the setting of progressive neurologic deficit, motor weakness, or severe neural compression [11,12]. In patients with preserved motor function, absence of bowel or bladder symptoms, and stable imaging findings, conservative treatment remains a reasonable initial approach [5]. In the present case, surgical decompression was deferred in favor of conservative care, and the addition of a targeted epidural steroid injection provided effective symptom relief.

## 4 Conclusion

This case underscores cement extravasation as a clinically significant complication of sacroplasty that may produce lumbosacral radiculopathy. Leakage may be influenced by fracture morphology, cortical disruption, cement viscosity, injection pressure, and needle trajectory, particularly in procedures performed near the sacral neural foramina. Although most cement leakages are asymptomatic, new dermatomal symptoms after sacroplasty or vertebroplasty should prompt immediate imaging evaluation. CT guidance may offer advantages over fluoroscopy by improving visualization of sacral anatomy and cement distribution, thereby facilitating earlier recognition of cement migration near neural structures. In carefully selected patients without progressive neurologic compromise, conservative treatment, including pharmacologic therapy, physical rehabilitation, and targeted epidural steroid injection, may achieve favorable outcomes. Surgical decompression remains indicated in the setting of progressive neurologic deficit, motor weakness, or severe neural compression.

## Ethical considerations

This case report was prepared as a retrospective chart review following completion of clinical care. No identifiable patient information is included. Institutional Review Board approval and written informed consent were not required.

## Use of technology

During the preparation of this case report, the author(s) used artificial intelligence to assist with language editing and organizational refinement. Following the use of this tool, the author(s) critically reviewed, edited, and revised the content and take full responsibility for the accuracy, integrity, and originality of the published work.
